# Engineering *Pseudomonas putida* To Produce Rhamnolipid Biosurfactants for Promoting Phenanthrene Biodegradation by a Two-Species Microbial Consortium

**DOI:** 10.1128/spectrum.00910-22

**Published:** 2022-06-22

**Authors:** Ruolin Qin, Tao Xu, Xiaoqiang Jia

**Affiliations:** a Department of Biochemical Engineering, School of Chemical Engineering and Technology, Tianjin University, Tianjin, People’s Republic of China; b Frontier Science Center for Synthetic Biology and Key Laboratory of Systems Bioengineering (MOE), School of Chemical Engineering and Technology, Tianjin University, Tianjin, People’s Republic of China; c Collaborative Innovation Center of Chemical Science and Engineering, Tianjin, People’s Republic of China; University of Minnesota

**Keywords:** PAHs, phenanthrene, engineered *Pseudomonas putida*, degradation, rhamnolipid, microbial consortium

## Abstract

Polycyclic aromatic hydrocarbons (PAHs) are a group of organic contaminants that pose a significant environmental hazard. Phenanthrene is one of the model compounds for the study of biodegradation of PAHs. However, the biodegradation of phenanthrene is often limited by its low water solubility and dissolution rate. To overcome this limitation, we engineered a strain of Pseudomonas putida to produce rhamnolipid biosurfactants and thereby promote phenanthrene biodegradation by an engineered strain of Escherichia coli constructed previously in our lab. The E. coli-P. putida two-species consortium exhibited a synergistic effect of these two distinct organisms in degrading phenanthrene, resulting in an increase from 61.15 to 73.86% of the degradation ratio of 100 mg/L phenanthrene within 7 days. After additional optimization of the degradation conditions, the phenanthrene degradation ratio was improved to 85.73%.

**IMPORTANCE** Polycyclic aromatic hydrocarbons (PAHs), which are recalcitrant, carcinogenic, and tend to bioaccumulate, are widespread and persistent environmental pollutants. Based on these characteristics, the U.S. Environmental Protection Agency has listed PAHs as priority contaminants. Although there are many methods to treat PAH pollution, these methods are mostly limited by the poor water solubility of PAHs, which is especially true for the biodegradation process. Recent evidence of PAH-contaminated sites suffering from increasingly severe impact has emerged. As a result, the need to degrade PAHs is becoming urgent. The significance of our study lies in the development of nonpathogenic strains of biosurfactant-producing Pseudomonas aeruginosa for promoting the degradation of phenanthrene by engineered Escherichia coli.

## INTRODUCTION

Polycyclic aromatic hydrocarbons (PAHs) are a class of recalcitrant organic chemicals with two or more aromatic rings (1), mainly derived from incomplete combustion of various organic compounds, including both natural and anthropogenic sources (2, 3). PAHs are widespread and can remain in the environment for many years leading to pollution in large areas (4). Moreover, PAHs are harmful to the environment and human health because of their potential carcinogenicity and mutagenicity (5). To address these PAH contamination issues, researchers have experimented with a variety of removal or *in situ* degradation strategies based on physical, chemical, and biological methods. Physical methods cannot remove PAHs completely and efficiently because they mostly transfer PAHs in space and do not change them chemically (6, 7). Using oxidants (8–10) to degrade PAHs is a common approach among chemical methods, which can solve the problems of time-consuming and inefficient physical methods. Biological methods have gained increasing attention since they overcome several drawbacks of chemical methods such as cost, procedural complexity, and secondary pollution (11). In contrast, biodegradation is considered to be safe, economical, and ecofriendly (12). Among all biological agents for PAH degradation, bacteria are considered especially promising (11, 13) due to their broad metabolic versatility for the biodegradation of PAHs (14), and most aerobic bacteria contain oxygenases that degrade PAHs (15). However, all of the methods described above, including biological methods, are limited by the low water solubility of PAHs. For example, when adding oxidants, the low aqueous solubility of PAHs limits the degradation efficiency (16). Regardless of the oxidizing agent used, the most critical factor for degradation was the poor availability of PAHs due to their low water solubility (17, 18). Similarly, as nonpolar compounds (6), PAHs are rarely soluble in aqueous media, which limits their bioavailability. Consequently, surfactant-mediated biodegradation was developed as a promising method for PAH degradation (11).

Many studies have added surfactants to increase the solubility of PAHs (19). Surfactants are amphiphilic molecules that contain a hydrophilic head and a hydrophobic tail (20). Surfactants can improve the solubility and bioavailability of PAHs by reducing surface tension and interfacial tension. They are generally classified into two types, including biosurfactants and synthetic surfactants. Compared to synthetic surfactants, biosurfactants showed better performance due to their biodegradability, low toxicity, and ability to facilitate direct transfer of PAHs to bacteria (11).

In this context, the application of rhamnolipids, mostly produced by Pseudomonas aeruginosa, successfully enhanced PAH degradation (21, 22). However, wild-type P. aeruginosa is an opportunistic pathogen, which limits its industrial and environmental applications. Moreover, the fermentation and purification of rhamnolipids is expensive, reaching $20 to $25 per kg, whereas synthetic surfactants cost $1 to $3 per kg (23). Therefore, heterologous expression of rhamnolipids in nonpathogenic strains such as Pseudomonas putida has become a research hot spot (24–27). Studies have shown that the *rhlAB* operon from P. aeruginosa is responsible for the biosynthesis of rhamnolipids, and the operon is regulated by the *rhlRI* genes, which are a part of the recombinant *rhlABRI* gene cluster. It had been shown that the overexpression of *rhlABRI* from P. aeruginosa EMS1 in P. putida KCTC led to the production of 7.3 g/L of rhamnolipids (28). Furthermore, heterologous production of rhamnolipids in P. putida KT2440 was achieved by replacing the native promoter of *rhlAB* with a synthetic promoter (29). These studies demonstrated the feasibility of producing rhamnolipids using P. putida.

Within the rapid development of emerging biological disciplines such as synthetic biology, increasing attention was paid to the design and development of artificial microbial consortia in order to overcome barriers in the degradation of PAHs. In previous research, our laboratory used a modular construction strategy to develop a consortium consisting of three engineered E. coli strains (30), whereby metabolic pathways were rationally segmented and allocated. Although this approach reduced the metabolic burden for individual cells and the consortium was able to degrade PAHs, the low water solubility was still a limiting factor for biodegradation. Here, we engineered P. putida as a heterologous host for rhamnolipid synthesis by expressing *rhlABRI* from P. aeruginosa and adding a copy of the strong promoter before each gene to enhance expression. Based on this strain, a two-species microbial consortium consisting of engineered E. coli and P. putida was formed that could efficiently degrade phenanthrene (PHE) in stable coculture. In addition, these two-species used phenanthrene and glycerol as carbon resources, respectively, thereby avoiding substrate competition among different bacteria and making full use of the substrates. Based on this system, we can further design more complex and efficient microbial consortia similar to wild communities for the efficient biodegradation of phenanthrene in polluted environments.

## RESULTS

### Design and construction of engineered *P. putida* producing rhamnolipid biosurfactants. (i) Heterologous gene expression.

**FIG 1:**
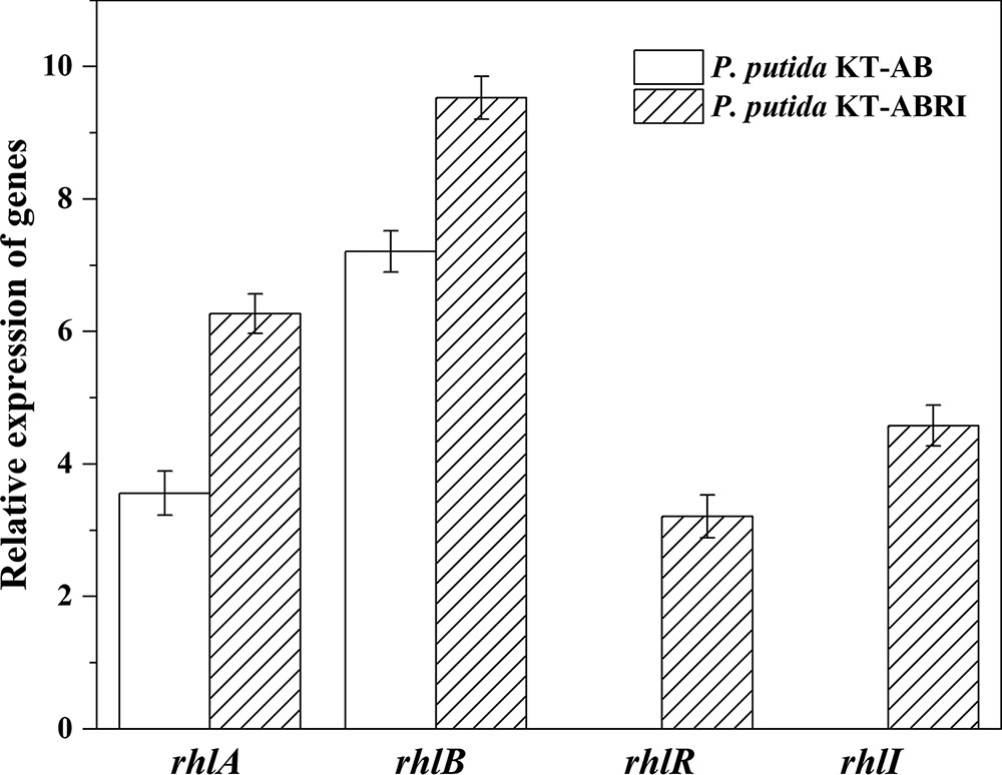
Relative expression multiple of genes (*rhlA*, *rhlB*, *rhlR*, and *rhlI*) for synthetic of rhamnolipid. These relative values were calculated compared to the expression of P. putida KT-159. The error bars represent standard deviations (*n* = 3).

We used real-time quantitative PCR (qRT-PCR) to determine the expression levels of the four heterologous genes *rhlABRI*. In contrast to the engineered P. putida KT-159 with the original promoter, the expressions of *rhlA* and *rhlB* were, respectively, 3.56- and 7.21-fold higher in engineered P. putida KT-AB, as well as 6.27- and 9.53-fold higher in P. putida KT-ABRI. Similarly, the expressions of *rhlR* and *rhlI* were, respectively, 3.21- and 4.58-fold higher in engineered P. putida KT-ABRI compared to P. putida KT-159 (Fig. 1).

### (ii) Growth of engineered *P. putida* and production of rhamnolipids.

**FIG 2:**
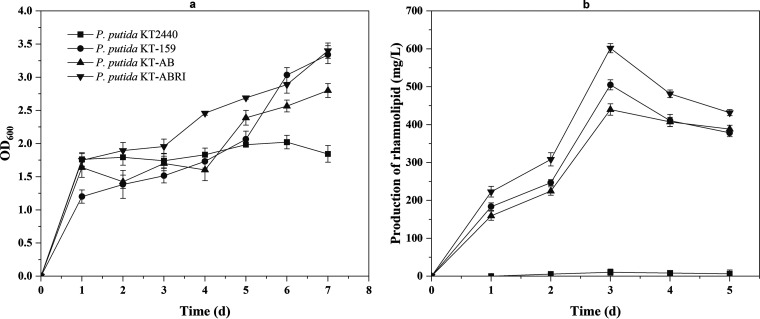
Performance of wild P. putida and engineered P. putida for the growth and production of rhamnolipid. (a) Growth of wild P. putida and engineered P. putida (P. putida KT-159, P. putida KT-AB, and P. putida KT-ABRI) in lipid-producing medium for 7 days. (b) Production of rhamnolipid with wild P. putida and engineered P. putida in lipid-producing medium for 5 days. The error bars represent standard deviations (*n* = 3).

To assess the growth of the engineered P. putida strains, we measured the optical density (OD) of the cultures in lipid-producing medium (Fig. 2a). The result shows that the heterologous gene expression had little effect on the growth of engineered strains in the first 4 days. Notably, the OD at 600 nm (OD_600_) was higher in engineered P. putida than in P. putida KT2440 after day 4. As expected, rhamnolipids were produced by the engineered P. putida in shake flasks using 3% glycerol as a carbon source. Trace amounts of rhamnolipids were also detected in the wild P. putida KT2440 due to a modified orcinol method (31) for the determination of rhamnolipid, and it was calculated from standard curves prepared with l-rhamnose expressed as rhamnose equivalents. Since rhamnose is widely contained in lipopolysaccharides on the cell surface of Pseudomonas spp., it is possible that some cells may have lysed during manipulation, resulting in a trace amount of rhamnolipids in the supernatant. The rhamnolipid titers of P. putida KT-159, P. putida KT-AB, and P. putida KT-ABRI reached maximum levels of 505.04, 439.85, and 601.7 mg/L on day 3, respectively (Fig. 2b).

### Design and construction of a synthetic two-species microbial consortium.

Our laboratory has successfully designed and constructed engineered E. coli M1, M2, and M3 following the principle of “division-of-labor” in previous work (see Table S1 in Text S1 in the supplemental material) (30). It should be noted here that we used these three engineered bacteria simultaneously in a 1:1:1 inoculation ratio in this study; this was referred collectively as E. coli M123. In this study, we further applied this principle to design three synthetic two-species microbial consortia composed of P. putida KT-159, P. putida KT-AB, or P. putida KT-ABRI and E. coli M123, which can degrade phenanthrene efficiently. The P. putida strains in the consortium can provide rhamnolipids for E. coli M123 to enhance the degradation of phenanthrene.

**FIG 3:**
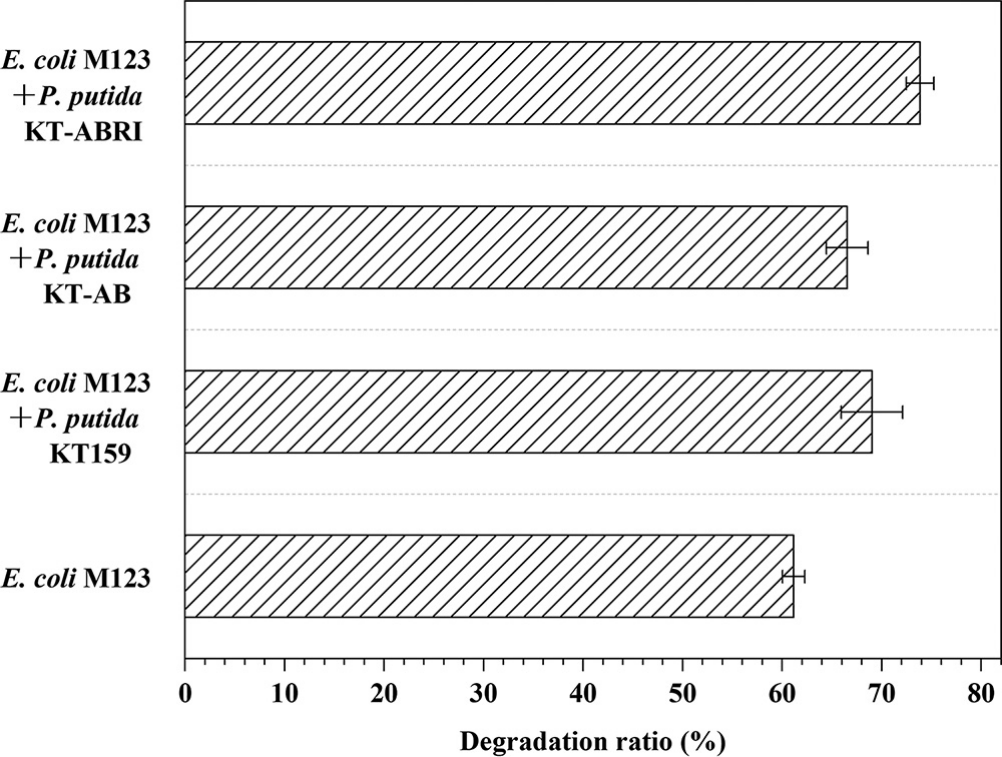
Degradation ratio of 100 mg/L phenanthrene with 7 days of four different consortia. The error bars represent standard deviations (*n* = 3).

In these synthetic microbial consortia, E. coli M123 and engineered P. putida, respectively, used phenanthrene and 3% glycerol as substrates to prevent competition. In order to verify the promoting effect of engineered P. putida and the degradation of the available phenanthrene by the synthetic consortium, the bacteria cultured in medium with 100 mg/L phenanthrene and 3% glycerol as carbon sources for 7 days. Generally, the degradation ratio of the consortia was higher than that of E. coli M123 alone over 7 days (Fig. 3). Specifically, the degradation ratio of the two-species consortium, including P. putida KT-ABRI, reached 73.86%, representing a 12.71% increase over E. coli M123 alone. Similarly, the degradation ratios of the consortia with E. coli M123 and P. putida KT-159 or P. putida KT-AB were promoted because of the addition of engineered P. putida. The concentration of phenanthrene in a culture consisting of E. coli M123 and P. putida KT-159 decreased consistently over 7 days, and 69.02% of the phenanthrene was degraded, compared to 66.54% in the consortium of E. coli M123 and P. putida KT-AB.

### Optimization of factors for phenanthrene biodegradation.

In order to improve the efficacy of the consortium, we further optimized the conditions, including IPTG (isopropyl-β-d-thiogalactopyranoside) inducer concentration, glycerol addition, and inoculation sequence. P. putida KT-ABRI was selected for further optimization based on its best degradation ratio of phenanthrene in preliminary experiments.

### IPTG inducer concentration.

**FIG 4:**
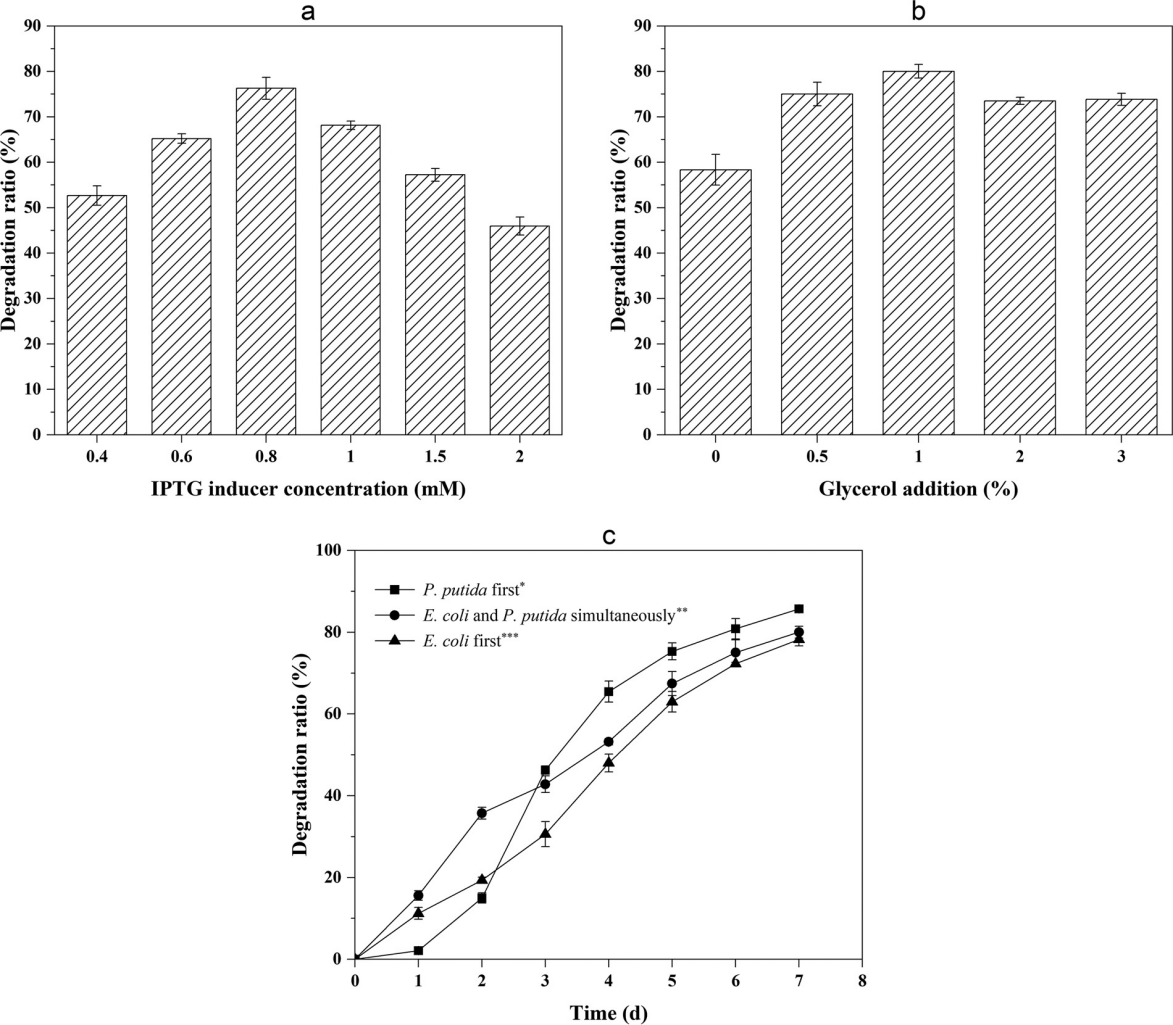
Condition optimization of degradation about three aspects. (a) Effect of IPTG addition on degradation ratio of 100 mg/L phenanthrene. (b) Effect of glycerol addition on degradation ratio of 100 mg/L phenanthrene. (c) Degradation ratio of different inoculation sequence. *, Inoculating engineered P. putida KT-ABRI first. **, Inoculating engineered P. putida KT-ABRI and E. coli M123 simultaneously. ***, Inoculating engineered E. coli M123 first. The error bars represent standard deviations (*n* = 3).

Here, the engineered E. coli M123 possess an IPTG-inducible promoter system, a *lac* operator, and a T7 promoter. Accordingly, we tested the influence of different IPTG concentrations on the degradation effect. The degradation ratio increased at IPTG concentrations between 0.4 and 0.8 mM, finally reaching the highest value of 76.28% (Fig. 4a). As the level of IPTG further increased, the degradation ratio stopped increasing and even decreased, indicating a strong metabolic burden due to strong protein expression or insoluble expression in inclusion bodies. The degradation effect was better at all IPTG concentrations than in the control without added IPTG, as expected.

### Glycerol addition.

The engineered P. putida uses glycerol as carbon source, and the glycerol concentration affects the yield of rhamnolipids, which in turn influences the phenanthrene degradation. Based on the first round of optimization, we further optimized the content of glycerol at an optimum concentration of IPTG. The degradation ratio increased as the amount of glycerol increased, and the highest value of 80.02% was obtained with the addition of 1% glycerol (Fig. 4b). However, at even higher glycerol concentrations of 2 and 3%, the degradation ratio decreased to 73.51 and 73.86%, respectively. Therefore, it was determined that the optimal content of glycerol was 1%, and subsequent experiments were carried out under this condition.

### Inoculation sequence.

Based on the first two optimized conditions (0.8 mM IPTG, 1% glycerol), we further explored the influence of the inoculation sequence of the synthetic consortium on the degradation ratio. The degradation ratio of the culture that was inoculated with P. putida KT-ABRI first was the highest, reaching 85.73% after 7 days (Fig. 4c). However, this inoculation sequence resulted in the slowest degradation of the three combinations during the initial 2 days. The degradation ratio after 7 days reached 78.22% with the consortium in which both species were added at the same time and reached 80.02% when E. coli M123 was added first. In particular, the degradation ratio of phenanthrene reached 35.74% after only 2 days with simultaneous inoculation. However, the ratio then decreased with the above inoculation method, while it consistently increased in the consortium inoculated with P. putida KT-ABRI first. Furthermore, these three strategies still maintained increasing degradation between 5 and 7 days (Fig. 4c).

### Remediation of soil contaminated with phenanthrene.

In order to test the suitability of the synthetic consortium for practical applications, five different strategies of remediation and two different concentrations of phenanthrene were tested in artificial phenanthrene-contaminated soils (Table 1).

**TABLE 1:** Different remediation strategies for phenanthrene-contaminated soils

Method	Remediation strategy
S0	Control with no treatment
S1	*E. coli* BL21 was inoculated in contaminated soil; soil moisture content: 15%
S2	*E. coli* M123 was inoculated in contaminated soil; soil moisture content: 15%
S3	*E. coli* M123 and *P. putida* KT-AB were inoculated in contaminated soil; 1% glycerol was added; soil moisture content: 15%
S4	*E. coli* M123 and *P. putida* KT-ABRI was inoculated in contaminated soil; 1% glycerol was added; soil moisture content: 15%

**FIG 5:**
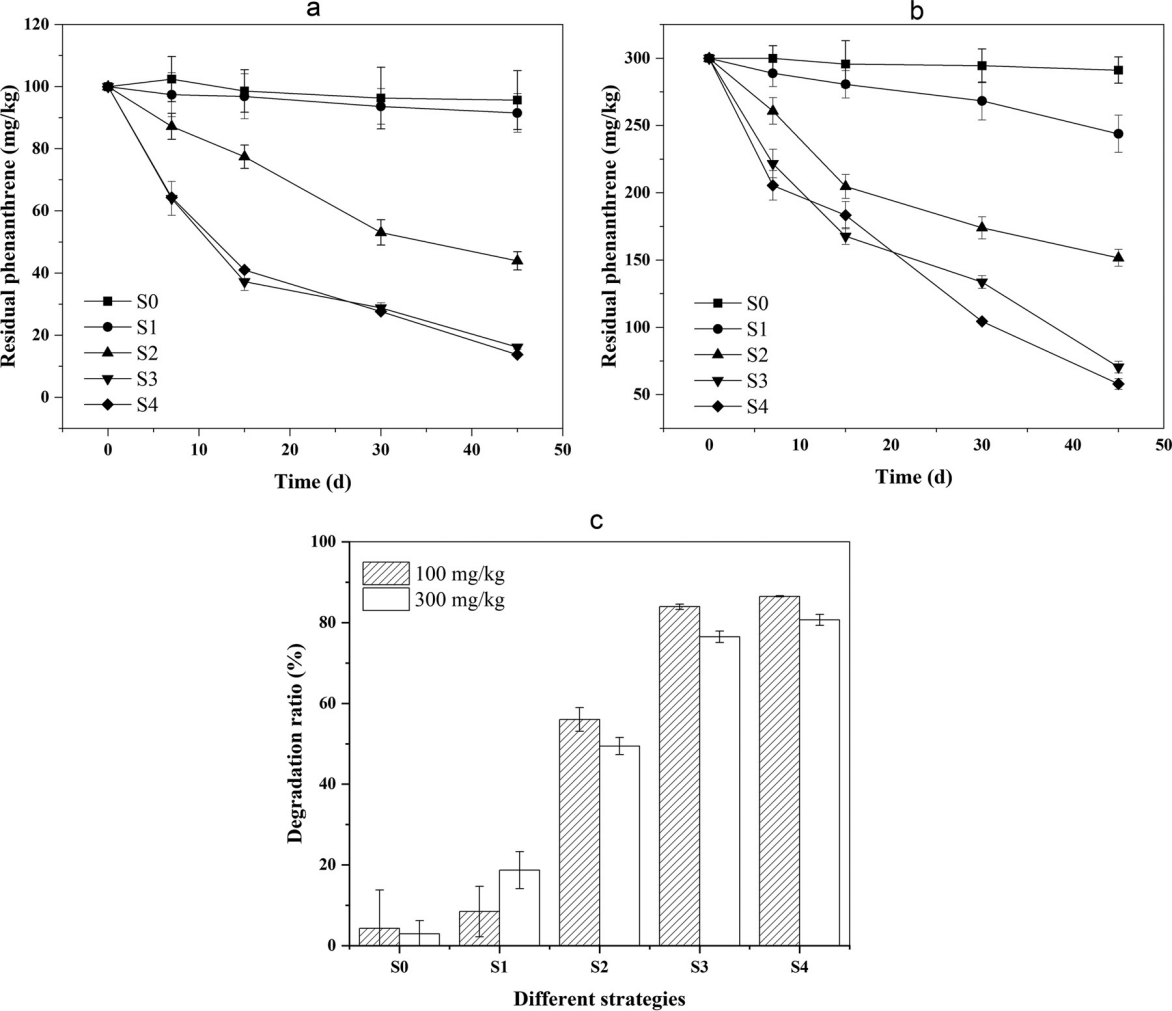
Degradation of PHE in different remediation strategies for 45 days. (a) The initial concentration of phenanthrene in soil is 100 mg/kg. (b) The initial concentration of phenanthrene in soil is 300 mg/kg. (c) The final degradation ratio in different remediation strategies after 45 days.

The residual contents of phenanthrene in soils were measured to test the removal efficiency and for phenanthrene tolerance of this consortium (Fig. 5). For method S0, the removal efficiency of phenanthrene was lowest, reaching 4.30 and 2.94% with 100 and 300 mg/kg of phenanthrene, respectively. Compared to S0, the phenanthrene removal efficiency of S1 was increased to 8.45 and 18.71%. When S2 was applied to remediate phenanthrene-contaminated soils, it was also limited by a lack of nutrients since it had no addition of glycerol that could be used as a carbon resource. Furthermore, the removal efficiency of phenanthrene reached to 83.84 and 86.52 at 100 mg/kg, while it was 76.49 and 80.67 at 300 mg/kg when S3 and S4 were applied. Among all these strategies, the phenanthrene removal efficiency of S4 was the highest, reaching 86.52% with the concentration of phenanthrene at 100 mg/L after 45 days.

## DISCUSSION

Rhamnolipids have many potential applications in industry, medicine, and bioremediation because of their low toxicity and biodegradability, combined with their potent reduction of surface tension and emulsifying activity (32). Rhamnolipids are mainly produced by P. aeruginosa with high productivity. However, P. aeruginosa is an opportunistic pathogen, which may cause health and safety concerns. In addition, phenanthrene is a hydrophobic compound, increasing the difficulties for biodegradation. In our study, we successfully designed and constructed an engineered P. putida for the production of rhamnolipids, which is a nonpathogenic bacterium. In P. aeruginosa, hydroxyalkanoyloxy-alkanoic acid (HAA) and dTDP-rhamnose are the two precursors required in the synthesis of rhamnolipids. RhlA is responsible for the formation of HAAs, followed by the synthesis of mono-rhamnolipids from dTDP-l-rhamnose and an HAA molecule in the presence of RhlB rhamnosyltransferase. The control of rhamnolipid production is complex, since it is influenced by numerous factors such as QS response (33). The QS response is dependent on the transcription activator RhlR and the autoinducer (AI) *N*-butyryl-homoserine lactone (C4-HSL) synthesized by RhlI. C4-HSL acts as the activating ligand of the transcriptional regulator RhlR, RhlR/C4-HSL complex and then binds to a specific sequence in the *rhlAB* regulatory region to activate the transcription. The production of rhamnolipids in P. putida KT-159 is consistent with the regulatory mechanism described above in wild P. aeruginosa, while transcription of the *rhlAB* gene is initiated by the Tac promoter in P. putida KT-AB. However, the mechanisms in P. putida KT-ABRI are not yet fully understood, especially the specific regulatory role of RhlR/I. Our hypothesis is that the self-inducible system RhlR/I not only activates the transcription of *rhlAB* by acting on the original promoter but also regulates the Tac promoter or other genes in P. putida. Further studies on the specific regulatory mechanism of KT-ABRI are in progress.

Based on previous literature, we amplified the *rhlABRI* rhamnolipid synthesis genes from Pseudomonas aeruginosa O-2-2. Furthermore, we changed the original promoter present in P. aeruginosa O-2-2 for the strong promoter Tac in the two recombinant vectors p2-rhlAB-Tac and p2-rhlABRI-Tac, with the aim of producing a vector with more efficient expression. The results confirmed that the vector with the Tac promoter (Fig. 1) and genes which are critical for rhamnolipid biosynthesis was successfully introduced into P. putida and expressed as expected, and the qRT-PCR analysis indicates that the replacement of the original promoter with Tac promoter played an important role. Similarly, by replacing the native promoter of *rhlAB* with a synthetic promoter with a two-phase feeding strategy, the rhamnolipid production reached 14.9 g/L in a previous study (29). It was previously reported that placing the *rhlAB* genes under the control of the Tac promoter in Burkholderia kururiensis increased the rhamnolipid titer to 5.76 g/L (34). In addition, the results showed that the expression of *rhlA* and *rhlB* increased after introducing *rhlR* and *rhlI*. The reason is that *rhlR* and *rhlI* are regulatory genes that control the synthesis of rhamnolipids (35). Accordingly, researchers produced 7.3 g/L of rhamnolipids by overexpressing *rhlAB* and *rhlRI* (36).

We also tested the growth and rhamnolipid production of the engineered P. putida strains in lipid-producing medium before attempting the construction of two-species synthetic consortia, including engineered P. putida. The strains exhibited good growth (Fig. 2a) and produced large amounts of rhamnolipids (Fig. 2b). Although other studies reported higher rhamnolipid titers (37, 38), smaller amounts are sufficient for biodegradation applications (39). It is noteworthy that the maximum titer in P. putida KT-AB was lower than the maximum titer produced by P. putida KT-159, which further demonstrates the importance of the *rhlR* and *rhlI* genes.

After having determined the growth and productivity of engineered P. putida, we cocultured the engineered P. putida with the previously constructed E. coli M123, which could degrade phenanthrene to build two-species microbial consortia. As previously mentioned, it should be noted that E. coli M123 is also a consortium consisting of E. coli M1, M2, and M3, which all have different metabolic functions (30). As anticipated, the three consortia containing engineered P. putida increased the degradation of 100 mg/L phenanthrene compared to E. coli M123 alone (Fig. 3). In these synthetic microbial consortia, especially for E. coli M123 and P. putida KT-ABRI, the degradation for phenanthrene was promoted by the rhamnolipids produced by the engineered P. putida, because our experiments showed that P. putida KT2440 and three engineered P. putida was unable to utilize phenanthrene no matter when it was the sole carbon source or when it was added with glycerol in mineral salt medium (MSM; data not shown). We have also tested several main intermediates of phenanthrene such as catechol, 1-hydroxy-2-naphthoic acid as the carbon source for P. putida, but it hardly grew, suggesting that P. putida does not consume these two major intermediates. However, the subsequent intermediates, including substances that can enter the tricarboxylic acid cycle, such as pyruvate and acetyl coenzyme A are definitely available to P. putida. We speculate that this may also be a facilitation mechanism for phenanthrene biodegradation with these two-species consortia. In addition, the degradation ratio was further improved in our synthetic two-species microbial consortium compared to exogenous addition of rhamnolipids, which suggested that our synthetic consortium benefited from the cooperation and mutualistic interactions which need to be further investigated among individual strains.

However, this effect was not significant compared to E. coli M123 alone, so we decided to further optimize the degradation conditions, including the IPTG inducer concentration, glycerol addition, and the inoculation sequence during the degradation process of phenanthrene to improve the degradation. First, there is a positive correlation between the concentration of IPTG and expression of genes in E. coli M123, but expression that is too strong can result in a metabolic burden that is too strong or insoluble expression in the form of inclusion bodies. Accordingly, the degradation ratio stopped increasing at IPTG concentrations above 0.8 mM and even decreased at inducer concentrations that were too high. In our previous study, the best degradation of phenanthrene was achieved by E. coli M123 with an IPTG concentration at 2 mM (30). However, as shown above, the optimized IPTG concentration for the two-species microbial consortia was 0.8 mM, since the engineered P. putida we constructed was not induced by IPTG in this work but instead had some toxic effects on the cells of engineered P. putida. Second, although rhamnolipids produced by engineered P. putida that used glycerol as a carbon source directly promoted phenanthrene degradation, the addition of glycerol was not as high as it could have been, and this promotion was weakened by concentrations of glycerol that were too high, as shown in Fig. 4b. Because glycerol has better bioavailability than phenanthrene, the synthetic consortium likely utilized glycerol more and only used phenanthrene as the sole carbon source when glycerol was exhausted. In addition, the rhamnolipids produced from the glycerol are also a possible secondary carbon source, leading to complex substrate competitions. Compared to the addition of 3% (vol/vol) glycerol before optimization, the degradation ratio of phenanthrene by the microbial consortium increased by 6.16% upon 1% glycerol addition. Our findings are in accordance with those of a previous study (39), which also found diverse effects on degradation at different glycerol concentrations. Finally, we tested the effect of the inoculation sequence of these two-species. The best degradation ratio reached 85.73% when P. putida was added first because the medium contains a certain amount of rhamnolipid after 24 h of inoculation with engineered P. putida that increases the solubility of phenanthrene in the medium due to the presence of rhamnolipid, which in turn increases the availability of phenanthrene to E. coli M123. Another reason is that the toxic effect of IPTG on engineered P. putida is somewhat attenuated by the fact that IPTG was added 6 h after inoculation with E. coli M123. However, the best sequence overall showed the lowest degradation ratio during the first 2 days. This is probably because the concentration of rhamnolipids was high, providing a carbon source that can be used instead of phenanthrene. The relatively low concentration of rhamnolipids could accelerate the dissolution of phenanthrene, and it had no apparent toxic effect on the cells as the concentration of phenanthrene decreased.

The highest degradation ratio of the consortium constructed in this study reached 85.73% of 100 mg/L after 7 days under the optimal conditions, while Thion et al. (40) reported a bacterial-fungal consortium that degraded 91% of 472 mg/kg phenanthrene within 46 days. Hence, the consortium exhibited good performance in terms of degradation of phenanthrene and can be used as a model for other PAH-degrading consortia.

Finally, we tested this two species microbial consortium in simulated contaminated soil to model practical remediation applications (Table 1). The control S0 method indicated that it is hard for indigenous microorganism in soils with a dramatic lack of water and nutrients to degrade phenanthrene (34). The degradation ratio with a moisture content of 15% was much higher, showing that suitable soil moisture could promote the degradation by indigenous microorganism. The experimental groups (S2, S3, and S4) showed significantly higher ratios of phenanthrene degradation compared to the control group (S0) without the addition of any consortia. In addition, S3 and S4 were 27.8 and 30.48% higher than S2, respectively, in the experimental group with a phenanthrene concentration of 100 mg/kg. The results indirectly suggested that our constructed engineered P. putida producing rhamnolipids can survive in the soil microcosms and promote the degradation of phenanthrene by E. coli M123 alone. Moreover, we tested the degradation of higher concentrations of phenanthrene (300 mg/kg) by the microbial consortia, and S4 (E. coli M123 + P. putida KT-ABRI) still degraded 80.67% of phenanthrene, indicating that the microbial consortia are tolerant to phenanthrene to some extent.

As a matter of fact, there are still some problems with our artificial consortia limiting applications in practice, such as the instability of plasmids and reliance on inducers and antibiotics, and we have taken these aspects into account. First, plasmid instability is an important concern, although the use of plasmids to transform exogenous degradation genes is a common operation in the biodegradation of contaminants. In addition, many degradation genes in nature are present in plasmids from wild strains (41). Regarding the specific plasmids used in this study, the high-copy vector pBBR1MCS-2 bearing the rhamnolipid production modules did not encounter any plasmid transmission loss during the experiments. However, we did take the instability of the plasmids into consideration, and when the consortia are applied in real environment, we can integrate the degradation genes into the chromosome by homologous recombination to increase the stability of gene expression. As for the addition of IPTG induction, the engineered P. putida KT-AB and KT-ABRI strains in our study are constitutively expressed, and the engineered P. putida KT-159 is controlled by RhlR/I system. The other member of the consortia, engineered E. coli M123, is required for IPTG induction. This problem can be avoided by replacing the IPTG-inducible T7 promoter with a constant promoter when needed (42). The use of antibiotics is to screen target strains in our experiments, they can be replaced by other nonantibiotic markers, such as fluorescent proteins (43), which can be used to determine the presence of target genes by detecting host luminescence when an artificial microbial consortium is used in a real environment. Alternatively, salt-tolerant genes can also be used as selection markers, for example, using *Halophilic* bacteria with low-cost sodium chloride as a tool for screening target strains (44). It should be noted that antibiotics were only used when culturing the strains in this study, and neither the degraded MSM nor the soil was supplemented with antibiotics after preparing the bacteriophage with an OD_600_ of ~3. We believe that the soil experiment is a good start and that practical applications need to be further explored and optimized.

## MATERIALS AND METHODS

### Plasmids, strains, genes, and primers.

Table S1 in the supplemental material lists the plasmids and strains. The primers used in this study are listed in Table S2. The sequences of *rhlA*, *rhlB*, *rhlR*, and *rhlI* from Pseudomonas aeruginosa O-2-2 are also provided in the supplemental material.

### Plasmid construction and transformation.

The cloned genes (*rhlA* and *rhlB*) related to the synthesis of rhamnolipids from Pseudomonas aeruginosa O-2-2 and the Tac promoter were inserted into the vector pBBR1MCS-2 (p2) to construct the expression plasmid p2-rhlAB-Tac. P. putida KT-AB was constructed by transforming the cells with the p2-*rhlAB*-Tac plasmid (see Fig. S1 in Text S1 in the supplemental material). Based on this, the cloned genes (*rhlR* and *rhlI*) and Tac promoter were inserted into the vector pBBR1MCS-2 (p2) to construct the expression plasmid p2-rhlABRI-Tac and P. putida KT-ABRI was constructed by transforming the cells with the p2-rhlABRI-Tac plasmid. Finally, the expression plasmid p2-*rhlABRI* was constructed by inserting the *rhlABRI* with the original promoter (see Fig. S1), resulting in the engineered P. putida KT-159 after transformation (see Fig. S1). E. coli M123 consisting of E. coli M1, M2, and M3 was stored in our lab.

### Culture media and culture condition.

Luria-Bertani (LB) medium contained the following (per L of dH_2_O):10 g peptone, 5 g yeast extract, and 5 g NaCl. The LB solid medium was added with extra 15 g/L agar. MSM contained the following (per L of dH_2_O): 5 g NaCl, 1 g NH_4_NO_3_, 1 g K_2_HPO_4_, 1 g KH_2_PO_4_, and 0.5 g MgSO_4_·7H_2_O. The lipid-producing medium contained the following (per L of dH_2_O): 2.5 g NaNO_3_, 4 g KH_2_PO_4_, 4 g K_2_HPO_4_, 0.1 g CaCl_2_, 0.4 g MgSO_4_·7H_2_O, 1 g NaCl, 1 g KCl, 1 g yeast extract, and 3% (vol/vol) glycerol.

E. coli M123 with 50 μg/mL streptomycin and the engineered P. putida, including P. putida KT-159, P. putida KT-AB, and P. putida KT-ABRI, with 50 μg/mL kanamycin were inoculated into LB broth shaking at 37°C and 30°C for 12 h, respectively. The growth rates were determined in the exponential growth phase.

### Quantitative real-time PCR.

The engineered P. putida (P. putida KT-159, P. putida KT-AB, and P. putida KT-ABRI) strains constructed in this study were inoculated into lipid-producing medium with 3% glycerol as the carbon source and then cultured overnight at 220 rpm at 30°C. These cultures were centrifuged at 12,000 rpm for 1 min to collect about 1 × 10^7^ cells for a single extraction. The RNA was extracted using a Total RNA Extraction kit. Quantitative real-time PCR was carried out according to a published method (45).

### Quantitative analysis of rhamnolipids.

In order to test the production of rhamnolipids in the wild-type strain P. putida KT2440 and engineered strains (P. putida KT-159, P. putida KT-AB, and P. putida KT-ABRI), three positive clones of each strain were cultured overnight at 30°C with shaking in LB broth with the addition of kanamycin. Rhamnolipid production was measured using the orcinol method. The concentration of extracellular glycolipids was evaluated by measuring the concentration of rhamnose (31). A sample comprising 333 μL of the culture supernatant was extracted three times with 1 mL of diethyl ether. After drying in an oven, it was resuspended in 0.5 mL of distilled water. Then, 200-μL samples were boiled with 0.19% orcinol and 53% H_2_SO_4_ for 20 min. After a cooling step at room temperature for 15 min, the absorbance values were measured at a wavelength of 421 nm, and the concentrations of rhamnolipid were calculated from standard curves prepared with l-rhamnose expressed as rhamnose equivalents.

### Determination of the content of phenanthrene in conical flasks.

The determination of phenanthrene during culture was conducted according to a published method (46). The integrals of the peak areas of pyrene and phenanthrene residues were used to calculate the phenanthrene degradation ratios. For removal estimations, three independent trials were carried out. The phenanthrene degradation ratio (DR) was calculated using the following formula:

$$\text{DR = }\frac{{\text{AF}}_{\text{control}}\;\text{–}\;\frac{{\text{AF}}_{\text{experimental}}\;\times \;{\text{AB}}_{\text{control}}}{{\text{AB}}_{\text{experimental}}}}{{\text{AF}}_{\text{control}}}\times \text{100\%}$$

The peak area of phenanthrene is denoted by AF, and the peak area of pyrene is denoted by AB.

### Preparation of artificially phenanthrene-contaminated soils.

In this study, 3.6 kg of original soil samples without contamination of phenanthrene was collected from Frontier Technology Research Institute of Tianjin University at a depth of 0 to 10 cm. After sieving, the soil was spiked using a phenanthrene solution of 100 mg/L. Then, half of the contaminated soil was divided into 12 plastic pots to ensure that each plastic pot contained 150 g of contaminated soil and that the final concentration of each was 300 mg/kg. The other half of contaminated soil was also divided into 12 pots, and the final concentration was 100 mg/kg. E. coli BL21, E. coli M1, E. coli M2, E. coli M3, P. putida KT-AB, and P. putida KT-ABRI were inoculated into LB liquid medium and cultured at 220 rpm at 30°C to the logarithmic growth stage. These cultures were centrifuged at 8,000 rpm for 10 min, and the supernatant was poured off. Then the organisms were washed three times with sterile water, after which a suspension at an OD_600_ of ~3 was prepared in phosphate-buffered saline. Portions (1 mL) of each of the suspensions described above were inoculated into each sample of contaminated soil (150 g). The moisture content of these samples was about 15%. The plastic pots were kept at 20 to 25°C.

### Remediation strategies for phenanthrene-contaminated soils.

We designed five strategies to remediate phenanthrene-contaminated soils (Table 1). These soil samples were stirred every week to supply oxygen, and the remediation was continued for 45 days. Finally, the residual phenanthrene content in the contaminated soils was determined as described below.

### Determination of phenanthrene content in soils.

Three samples each containing 10 g of phenanthrene-contaminated soil were taken from each pot after 7, 15, 30, and 45 days, and the total PAHs were extracted as follows. First, a 10 g sample was placed into a conical 150 mL flask. Then, 25 mL of *n*-hexane was added to the flask and shaken gently to mix the *n*-hexane and soil particles thoroughly. Next, the sample was left to stand for 3 min, and the supernatant was then transferred into a beaker. This step was repeated once, followed by analysis according to a published method (46).
