# Supplementary material for: Engineering Pseudomonas putida To Produce Rhamnolipid Biosurfactants for Promoting Phenanthrene Biodegradation by a Two-Species Microbial Consortium
Source: Microbiol Spectr. 2022 Jun 22;10(4):e00910-22. doi: 10.1128/spectrum.00910-22 (PMC9431653; doi:10.1128/spectrum.00910-22)
Supplement: Supplemental file 1 — Supplemental material. Download spectrum.00910-22-s0001.pdf, PDF file, 0.3 MB [file spectrum.00910-22-s0001.pdf]

Engineering *Pseudomonas putida* to Produce Rhamnolipid Biosurfactants for Promoting Phenanthrene Biodegrading by a Two-species Microbial Consortium

Ruolin Qin[a], Tao Xu[a], Xiaoqiang Jia[a,b,c]#

[a]Department of Biochemical Engineering, School of Chemical Engineering and Technology, Tianjin University, Tianjin, PR China

[b]Frontier Science Center for Synthetic Biology and Key Laboratory of Systems Bioengineering (MOE), School of Chemical Engineering and Technology, Tianjin University, Tianjin, PR China

[c]Collaborative Innovation Center of Chemical Science and Engineering (Tianjin), Tianjin, PR China

Running title: Engineering *P. putida* for Promoting PHE Biodegrading

#Address correspondence to Xiaoqiang Jia, xqjia@tju.edu.cn.

Ruolin Qin and Tao Xu contributed equally to this work. Author order was determined on the basis of contribution.

Key words: PAHs; phenanthrene; engineered *P.putida*; degradation; rhamnolipid; microbial consortium

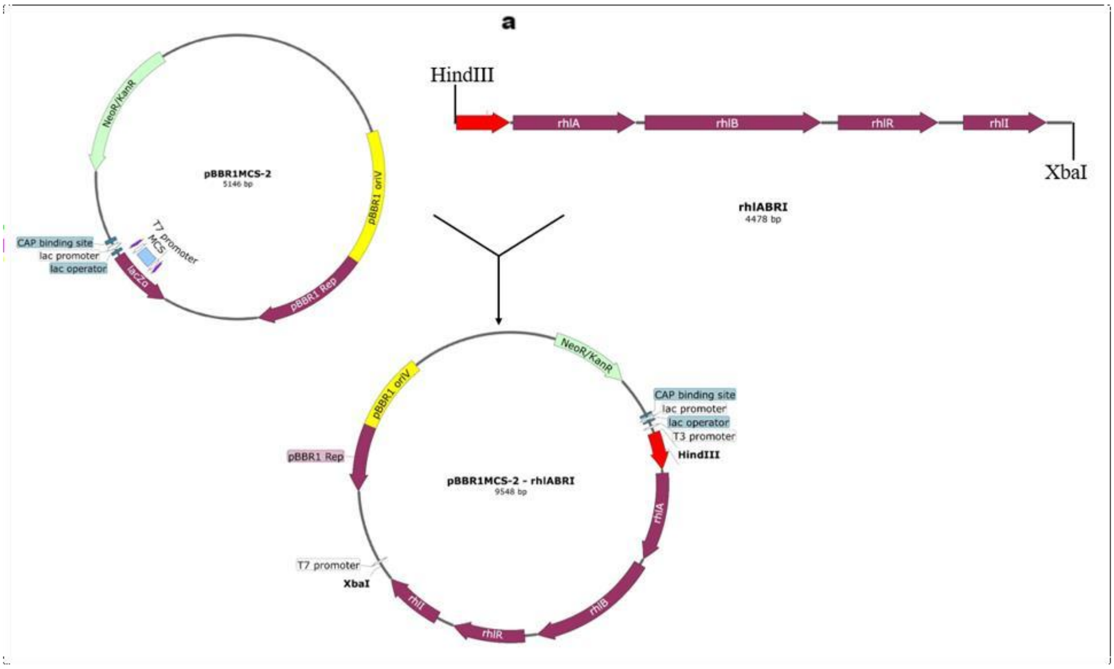
a
HindIII
rhlA
rhlB
rhlR
rhlI
XbaI
rhlABRI
4478 bp
pBBR1MCS-2
5146 bp
NeoR/KanR
pBBR1 oriV
pBBR1 Rep
lacZα
MCS
T7 promoter
CAP binding site
lac promoter
lac operator
pBBR1MCS-2 - rhlABRI
9548 bp
T3 promoter
HindIII
T7 promoter
XbaI

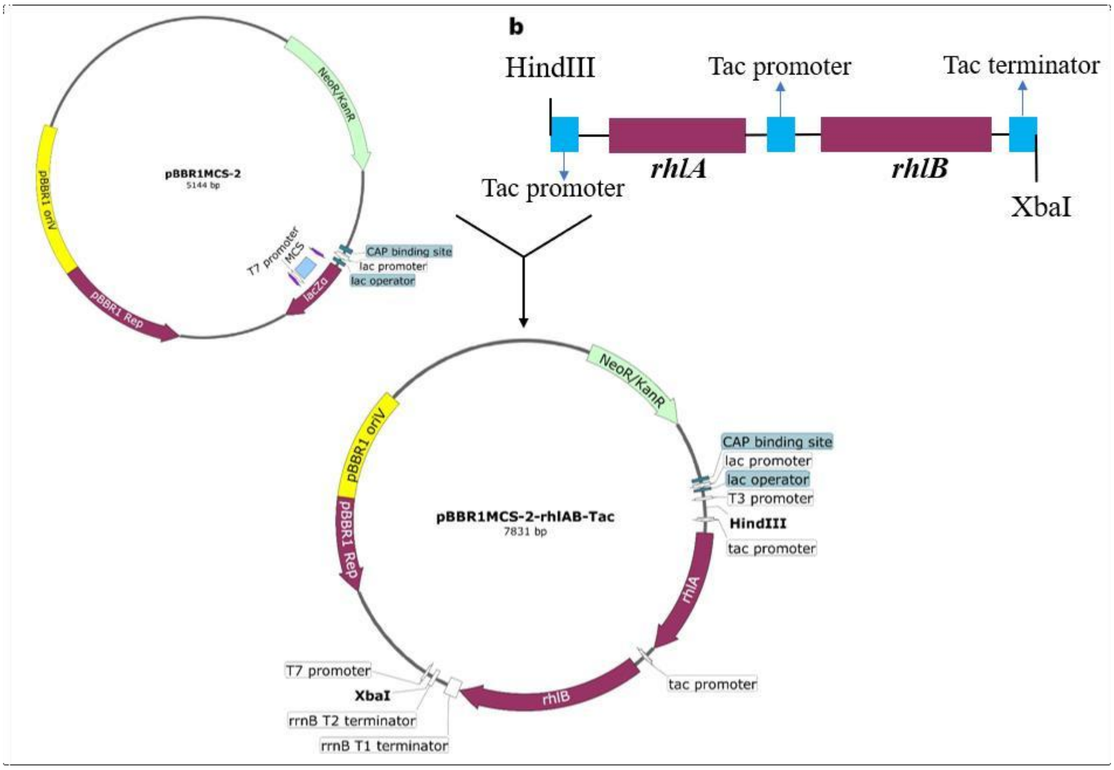
b
HindIII
Tac promoter
Tac terminator
Tac promoter
rhlA
rhlB
XbaI
pBBR1MCS-2
5144 bp
NeoR/KanR
pBBR1 oriV
pBBR1 Rep
lacZα
MCS
T7 promoter
CAP binding site
lac promoter
lac operator
pBBR1MCS-2-rhlAB-Tac
7831 bp
T3 promoter
HindIII
tac promoter
tac promoter
T7 promoter
XbaI
rrnB T2 terminator
rrnB T1 terminator

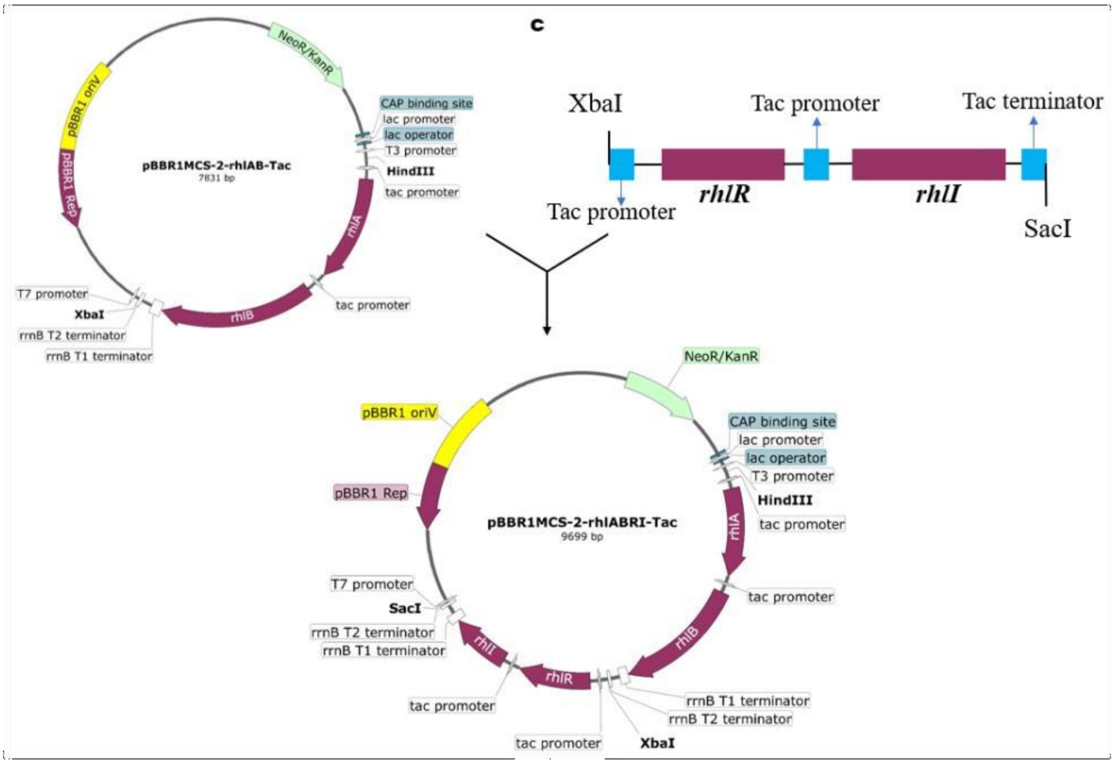

FIG S1 Outline of the plasmid construction strategy. The genes *rhlA* and *rhlB* indicate the rhamnosyltransferase chain A and rhamnosyltransferase chain B, respectively. The genes *rhlR* and *rhlI* encode the N-butyrylhomoserine lactone autoinducer synthase. (a) The construction of pBBR1MCS-2-rhlABRI. (b) The construction of pBBR1MCS-2-rhlAB-Tac. (c) The construction of pBBR1MCS-2-rhlABRI-Tac.

TABLE S1 Bacterial strains and plasmids used in this study.

Name	Relevant characteristics	Source
**Plasmids**		
pCDFDuet-1	double T7, Lac; Str^r	Miaoling Biotech
pM1	pCDFDuet-1 with *phdF, phnD, phdG, nidD* and *phnF*	Laboratory stock
pM2	pCDFDuet-1 with *nahC-G*	Laboratory stock
pM3	pCDFDuet-1 with *catA-D*； *pcaI*, *pcaJ* and *pcaF*	Laboratory stock
pBBR1MCS-2	PBBR1 oriV with P_{T7}; Kan^R	Laboratory stock
p2-*rhlABRI*	pBBR1MCS-2 with *rhlABRI* operon	This study
p2-*rhlAB*-Tac	pBBR1MCS-2 with *rhlAB* operon and *Tac* promoter	This study
p2-*rhlABRI*-Tac	pBBR1MCS-2 with *rhlABRI* operon and *Tac* promoter	This study
**Strains**		
E coli BL21(DE3)	F– ompT hsdSB(rB^- mB^-) gal dcm (DE3)	TransGen Biotech
E coli M1	*E. coli* BL21 with pM1	Laboratory stock
E coli M2	*E. coli* BL21 with pM2	Laboratory stock
E coli M3	*E. coli* BL21 with pM3	Laboratory stock
P. aeruginosa O-2-2	Wild type strain; used for amplification of *rhlABRI* operon	Laboratory strain
P. putida KT2440	Wild type strain	Laboratory strain
P. putida KT-159	*P. putida* KT2440 with p2-*rhlABRI*	This study
P. putida KT-AB	*P. putida* KT2440 with p2-*rhlAB*-Tac	This study
P. putida KT-ABRI	*P. putida* KT2440 with p2-*rhlABRI*-Tac	This study

TABLE S2 Primers used in this study.

Primers	Sequence	Characteristics
rhlA-HindIII-F	CCCAAGCTTTTCGACACCGGAAACCGGGC	27 bp, HindIII
rhlI-XbaI-R	GCTCTAGAGGCATGGCGACTCCTCGGG	27 bp, XbaI
rhlA-F	TAGATGCGTCGCGAGAGCC	19 bp
rhlA-R	GGTCAGGCGTAGCCGATGG	19 bp
rhlB-F	ATGCACGCCATCCTGATCG	19 bp
rhlB-R	TCAGCTGGCGGCTTTCAG	18 bp
rhlR-F	TAGATGCGCAACGACGGTG	19 bp
rhlR-R	TCAGATCAGACCCAGCGCG	19 bp
rhlI-F	ATGATCGAGCTGCTGAGCGA	20 bp
rhlI-R	TCACACGGCCATGCTCAGC	19 bp
**qPCR**		
rhlA-F	GAACGAAACCGTGGGCAA	18 bp
rhlA-R	GGACCTGGTCGATGTGGAAA	20 bp
rhlB-F	TGACCTACCGTCGCACCAT	19 bp
rhlB-R	GGGATGCCGTACTTCTCGTG	20 bp
rhlR-F	TTCACCCGTCCGAAAACC	18 bp
rhlR-R	AACAGCGAGTCCGACCACAC	20 bp
rhlI-F	GCGCGACCAAGAATTCGA	18 bp
rhlI-R	TGCTGGCACCCAGATACCA	19 bp

[a]The restriction sites on the primers are indicated by underscore.
